# Irritability in ADHD: association with later depression symptoms

**DOI:** 10.1007/s00787-019-01303-x

**Published:** 2019-03-05

**Authors:** Olga Eyre, Lucy Riglin, Ellen Leibenluft, Argyris Stringaris, Stephan Collishaw, Anita Thapar

**Affiliations:** 1 MRC Centre for Neuropsychiatric Genetics and Genomics, Division of Psychological Medicine and Clinical Neurosciences, Cardiff University, Hadyn Ellis Building, Maindy Road, Cardiff, CF24 4HQ UK; 2 Emotion and Development Branch, National Institute of Mental Health, Bethesda, MD USA

**Keywords:** ADHD, DMDD, Irritability, Depression

## Abstract

Attention-deficit/hyperactivity disorder (ADHD) and depression commonly co-occur. Identifying children with ADHD at risk for later depression may allow early intervention and prevention. Irritability is one possible mechanism linking these two disorders. It is common in ADHD and associated with later depression in the general population. Cross-sectional studies suggest an association between irritability and depression in ADHD, but longitudinal research is limited. This study followed up a clinical ADHD sample longitudinally to examine: (1) the association between childhood irritability and later depression symptoms, and (2) whether irritability persistence is important in this association. At baseline, parents (*n *= 696) completed semi-structured interviews about their child (mean age = 10.9), providing information on child psychopathology, including irritability. A subsample (*n *= 249) was followed up after a mean of 5.4 years. Parent-completed Mood and Feelings Questionnaires provided information on depressive symptoms at follow-up. Parent-rated structured diagnostic interviews provided information on ADHD diagnosis and irritability at follow-up. Regression analyses examined associations between (i) baseline irritability and depression symptoms at follow-up, and (ii) persistent (vs. remitted) irritability and depression symptoms at follow-up. Analyses controlled for age, gender, depression symptoms, anxiety, ADHD symptoms, and ADHD medication at baseline. Baseline irritability was associated with depression symptoms at follow-up, but the association attenuated after controlling for anxiety and ADHD symptoms. Persistent irritability was associated with depression symptoms at follow-up, after including all covariates. Children with ADHD with persistent irritability are at elevated risk of developing depression symptoms. They may be a target for early intervention and prevention of depression.

**Electronic supplementary material:**

The online version of this article (10.1007/s00787-019-01303-x) contains supplementary material, which is available to authorized users.

## Introduction

Attention-deficit/hyperactivity disorder (ADHD) is a common, impairing neurodevelopmental disorder characterised by inattention, hyperactivity and impulsivity. Comorbidity is common, with more than 50% experiencing at least one other psychiatric disorder [1, 2]. Major depressive disorder (MDD) has been observed to occur more frequently in young people with ADHD than in those without [3–5], and levels of depression symptoms are higher across young adulthood in those with a history of childhood ADHD than in those without the disorder [6]. A meta-analysis found rates of MDD to be on average more than five times higher in those with ADHD than in those without [7].

This is important, as when depression co-occurs with ADHD, outcomes are worse than for either disorder alone. There is increased psychosocial impairment [8] and elevated risk for psychiatric hospital admission and suicidality [3]. In addition, as ADHD precedes the onset of depression [9], identifying those at risk provides an opportunity for early intervention and prevention.

Children with ADHD who are also irritable may be an important at-risk group for future depression. That is because population-based studies have consistently found irritability to be associated longitudinally with depression [10]. Irritability is a propensity to react with anger, grouchiness or tantrums disproportionate to the situation [11]. When severe, irritability is impairing and is a common reason for referral to child psychiatry services [12]. It is particularly common in those with ADHD [13] and thus a possible mechanism that explains the high rates of depression in this group.

There has been little research into the longitudinal association between irritability and depression in those with ADHD. Although findings from the general population suggest an association, these cannot automatically be extrapolated to young people with ADHD. Firstly, it is not clear whether the irritability observed frequently in ADHD is the same as the irritability measured in the general population. Secondly, it is possible that gender may impact on the association between irritability and depression, and children with ADHD are predominantly male.

To date, two cross-sectional clinical ADHD samples have examined the association between irritability and depression, finding that irritability symptoms and the DSM-5 diagnosis of disruptive mood dysregulation disorder (DMDD), characterised by severe temper outbursts and persistent irritability, were associated with depression symptoms [14, 15]. Longitudinally, the association between irritability and depression in a clinical ADHD sample is yet to be examined. Seymour et al. [16] did utilise a population sample, finding evidence that emotion regulation (a broader construct than irritability) mediates the relationship between ADHD symptoms and later depression symptoms. However, longitudinal investigation in a clinical ADHD sample is needed.

It is also relevant to consider whether irritability persistence is important in the relationship between irritability and depression. Understanding whether it is persistent rather than remitting irritability that confers the greatest risk of depression would allow more precise targeting of preventive interventions. Pagliaccio et al. [17] found that, in a sample enriched for preschool depression, those with consistently elevated irritability trajectories across childhood were more likely to develop depression than those whose levels of irritability started high but decreased over time. Wiggins et al. [18] also derived irritability trajectories across childhood (age 3–9 years), finding that internalising symptoms generally mirrored the patterns of the irritability trajectories (children in a high, steady irritability trajectory or high, increasing irritability trajectory had higher internalising symptoms by age 9 years than those with initially high but decreasing irritability). However, the impact of persistent irritability on risk for depression in children with ADHD has not been examined.

The aims of this study were to utilise an ADHD patient sample that was longitudinally assessed to: (1) examine the association between childhood irritability and adolescent depression symptoms, and (2) examine whether irritability persistence (vs. remittance) is important in this association.

## Methods

### Sample

The sample was made up of children who previously took part in the Study of ADHD, Genes and Environment (SAGE), at Cardiff University, UK. SAGE is a study of 696 children aged 6–18 years (mean 10.9 years, SD = 2.99) who were recruited through psychiatry and paediatric clinics between 2007 and 2011 [19]. All had a clinical diagnosis of ADHD and met DSM-IV or DSM-III-R research diagnostic criteria for ADHD. 84% were male and had a mean IQ of 83. All were of British Caucasian origin and living with at least one biological parent at the time of the study. Children were excluded if they had any major comorbid neurological disorder, psychosis, Tourette’s syndrome, autism or genetic syndrome.

**Fig. 1:**
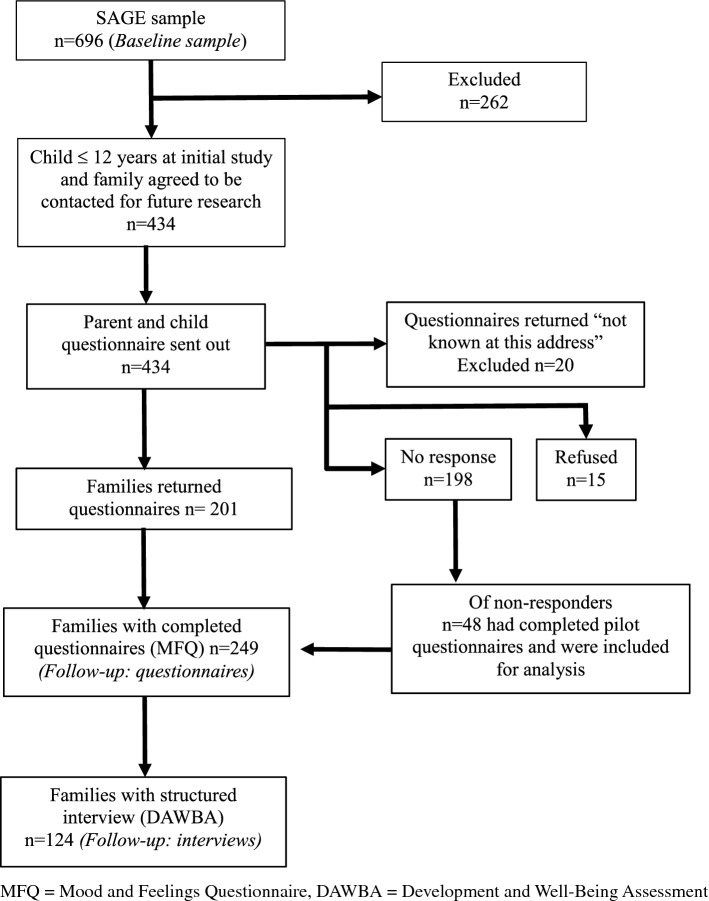
Flowchart showing numbers completing follow-up questionnaires and interviews

A subsample of these participants aged ≤ 12 years at the time of initial data collection (mean age = 9.2 years, SD = 1.95), and whose family had consented to be contacted for future research, was invited to take part in a follow-up study. The age criteria for inclusion (≤ 12 years at the time of initial data collection) were chosen to limit the prevalence of depression in the children at baseline. Follow-up was on average 5.4 years after initial participation (range 2–9 years, SD = 1.42). A total of 434 eligible participants were sent follow-up postal questionnaires between 2013 and 2016. 249 families returned completed questionnaires. Therefore, follow-up data were available for 57% of the eligible sample. Of those completing questionnaires, 124 also completed structured diagnostic interviews at follow-up. Figure 1 provides a flowchart detailing the numbers of participants taking part at each stage of the study. Ethical approval for this study was obtained from the Wales Multi-Centre Research Ethics committee. Parents provided written informed consent and children gave written assent.

### Measures

#### Baseline assessment (time 1)

##### Child psychopathology

The parent-completed Child and Adolescent Psychiatric Assessment (CAPA) was used to measure baseline psychopathology in the child. The CAPA is a semi-structured diagnostic interview that involves interviewers asking about symptoms of a range of psychiatric disorders present in the last 3 months [20]. The parent-completed CAPA was used to ascertain the presence of DSM-5 psychiatric disorders including ADHD, major depressive disorder (MDD) and common childhood anxiety disorders (generalised anxiety disorder and separation anxiety disorder). Symptom counts for MDD and ADHD were also derived based on the information provided in the CAPA. For symptoms to be endorsed on the CAPA they must be uncontrollable and interfere with at least two activities. All interviewers were trained to a high level of reliability. All interviews were recorded, and interviewers were supervised weekly by an experienced child and adolescent psychiatrist (AT).

Irritability: The CAPA was also used to assess baseline irritability. Irritability was defined both as a continuous score and a categorical diagnosis. The irritable score was calculated using three items from the oppositional defiant disorder (ODD) section of the CAPA, previously defined as making up an irritable dimension of ODD [21]. The items were “temper tantrums”, “touchy/easily annoyed” and “angry or resentful”. A total score of 0–3 was generated based on the presence or absence of these items. A categorical DSM-5 diagnosis of disruptive mood dysregulation disorder (DMDD) was derived using items from the depression and ODD sections of the CAPA (described in Supplementary Material Table S1). We focused on DMDD diagnosis rather than ODD diagnosis as we were interested specifically in the association between irritability (rather than non-irritable ODD symptoms) and depression given previous findings [21–23].

##### Other measures

Demographic information was recorded on parent-completed questionnaires at baseline. Child ADHD medication status was recorded using the parent-completed CAPA. Parents were asked whether or not their child takes stimulant medication. All children completed the WISC-IV [24], providing a full-scale IQ.

#### Follow-up assessment (time 2)

##### Child psychopathology: questionnaire data

Depression symptoms: The parent-completed Mood and Feelings Questionnaire (MFQ) [25] was the primary measure used to measure depression symptoms at follow-up. The parent-completed MFQ is a widely used depression screening instrument [26]. It is made up of 34 items, each rated as 0 (not true), 1 (sometimes true) and 2 (true). Item scores were used to derive a total score, with a possible range of 0–68. A score of ≥ 21 on the parent-rated MFQ is an accepted cutoff when screening for possible depression [26] and was used to generate a binary MFQ outcome measure.

The child self-rated Mood and Feelings Questionnaire (MFQ) was also completed at follow-up on a smaller sample of participants (*n *= 169). The child self-rated MFQ is also widely used as a depression screening instrument [26] and is made up of 33 items (same items as the parent report, except “s/he wasn’t as happy as usual, even when s/he was praised or rewarded” is not included). It has a possible score range of 0–66.

Previous findings have suggested that young people with ADHD may under-report their own depression symptoms [27] which is why parent-reported depression was the primary outcome.

##### Child psychopathology: interview data

The parent-completed Development and Well-Being Assessment (DAWBA) was used to measure child psychopathology at follow-up. The DAWBA is a structured interview covering common emotional, behavioural and hyperactivity disorders [28]. For each diagnostic category, computerised DAWBA algorithms can be used to generate probability bands, ranging from a probability of having the relevant diagnosis of less than 0.1% to > 70%. The two highest probability bands have been described as equivalent to clinician-rated diagnosis [29]. These were used to establish the presence of DSM-5 ADHD and MDD at follow-up. ADHD persistence was defined as presence of ADHD diagnosis at follow-up (as all of the sample had ADHD at baseline).

Irritability: Irritability at follow-up was also assessed using the DAWBA. A continuous score was generated using the same three items from the ODD section that were used to make the irritability score from the CAPA at baseline. They included “temper outbursts”, “easily annoyed” and “angry and resentful”. If the item was rated as being present “rarely or never” or “at least once per week”, then a score of 0 was assigned for that item. If it was rated as being present “most days” or “every day” then a score of 1 was assigned, providing a possible total score of 0–3. Irritability persistence was defined as an irritable score of ≥ 1 on the CAPA at baseline and ≥ 1 at follow-up on the DAWBA. A categorical diagnosis of DMDD was derived based on the symptoms reported in the DMDD section of the DAWBA (described in Supplementary Material Table S1).

### Analyses

Analyses were carried out using Stata version 14.

The baseline (time 1) characteristics and rates of psychopathology for those who took part at each stage of the study were described, allowing comparison of responders and non-responders at follow-up. Follow-up (time 2) characteristics and rates of psychopathology were described for those who completed follow-up questionnaires/interviews.

#### Association between irritability at baseline and later depression symptoms

The mean irritability score and percentage meeting criteria for DMDD diagnosis at baseline were established. Total parent-rated MFQ scores and total child self-rated MFQ scores at follow-up were calculated. Where > 3 MFQ items (> 10%) were missing for an individual, the total score was counted as missing. Where ≤ 3 (< 10%) MFQ items were missing, a mean score of the completed items was imputed.

Linear regression was carried out to examine the association between childhood irritability (baseline irritability score and DMDD diagnosis) and adolescent depression symptoms (follow-up parent-rated MFQ total). Due to the small numbers of participants who met diagnostic criteria for major depressive disorder on the DAWBA at follow-up (*n *= 6), it was not possible to examine the association between irritability and major depressive disorder diagnosis. Instead, a binary MFQ measure, based on being above or below the clinical cut point of ≥ 21 on the parent-rated MFQ, was used as an outcome. Regression analyses were run unadjusted, then controlling for child age, gender and baseline depression symptoms. Further covariates were assessed as sensitivity analyses (see below).

#### Persistent vs. remitted irritability

The percentage of the sample with persistent irritability was established. Linear regression was used to examine whether persistent vs remitted irritability was associated with total parent-rated MFQ score at follow-up, as well as examining the association between persistent vs remitted irritability and the binary MFQ outcome. Analyses were run, first unadjusted, then controlling for child age, gender and baseline depression symptoms. Further covariates were again assessed in sensitivity analyses (see below).

#### Sensitivity analyses

Sensitivity analyses were conducted by (i) controlling for DSM-5 diagnosis of anxiety disorder (as anxiety commonly co-occurs with irritability and is associated with depression), ADHD symptoms and medication status at baseline (in addition to age, gender and baseline depression symptoms), (ii) removing the irritable item (he/she felt grumpy and irritable with his/her parents) from the parent-rated MFQ at follow-up, (iii) examining whether any association between persistent irritability and depressive symptoms was explained by persistent ADHD and (iv) using child self-rated MFQ total score to measure depression symptoms at follow-up.

## Results

### Sample characteristics

The baseline characteristics of participants who took part at each stage of the study are described in Table 1. Those who took part in follow-up were younger than those who were eligible but did not take part (9.0 vs 9.5 years, *t*(432) = 2.45, *p *= 0.01). However, there were no significant differences between responders and non-responders in terms of gender, IQ, parental income, ADHD medication or baseline child psychopathology (Supplementary Table S2). Sample characteristics and rates of psychopathology at follow-up are reported in Table 2.

**Table 1:** Baseline characteristics of (i) those taking part at baseline, (ii) those invited to follow-up, (iii) those completing follow-up questionnaires and (iv) those completing follow-up interviews

	(i) SAGE sample (*n *= 696)^a^	(ii) Invited to follow-up (*n *= 434)^b^	(iii) Follow-up: questionnaire (*n *= 249)^c^	(iv) Follow-up: interview (*n *= 124)^d^
Gender, % male (*n*)	84% (583)	82% (358)	82% (204)	80% (99)
Age, in years (range, SD)	10.9 (6–18, SD = 2.99)	9.2 (6–12, SD = 1.95)	9.0 (6–12, SD = 1.90)	8.5 (6–12, SD = 1.80)
IQ (range, SD)	83 (41–119, SD 13.4)	84 (46–119, SD = 12.4)	85 (50–119, SD = 12.5)	84 (58–118, SD = 12.0)
Income, % < £20,000/year (*n*)	63% (358)	66% (239)	62% (133)	68% (74)
ADHD medication, % (*n*)	80.6% (554)	77.3% (333)	77.9% (194)	79.0% (98)
Irritability score, mean (range, SD)	2.19 (0–3, SD = 1.0)	2.24 (0–3, SD = 0.95)	2.22 (0–3, SD = 0.94)	2.38 (0–3, SD = 0.79)
DMDD diagnosis, % (*n*)	31% (207)	37.2% (152)	39.2% (93)	45.8% (55)
Anxiety disorder % (*n*)	6.1% (40)	7.3% (30)	7.9% (19)	10.7% (13)
MDD diagnosis, % (*n*)	1.9% (13)	1.4% (6)	1.6% (4)	0.8% (1)

*ADHD* attention-deficit/hyperactivity disorder, *DMDD* disruptive mood dysregulation disorder, *MDD* major depressive disorder, *Anxiety disorder* includes generalised anxiety disorder or separation anxiety disorder, DMDD anxiety disorder and MDD diagnoses made using the CAPA, based on DSM-5 diagnostic criteria

^a^Number available for each variable ranged from 565 to 696

^b^Number available for each variable ranged from 364 to 434

^c^Number available for each variable ranged from 214 to 249

^d^Number available for each variable ranged from 109 to 124

**Table 2:** Characteristics of respondents at follow-up

	Follow-up: questionnaires (*n *= 249)^a^	Follow-up: interviews (*n *= 124)^b^
Gender, % male (*n*)	82% (204)	81% (99)
Age, in years (range, SD)	14.4 (8–19, SD = 2.38)	14.7 (11–20, SD = 2.10)
ADHD medication, % (*n*)	69.6% (126)	69.8% (81)
MFQ total score (range, SD)	24.4 (0–68, SD = 15.4)	23.7 (0–68, SD = 15.13)
ADHD diagnosis, % (*n*)	–	66.7% (82)
Irritability score (range, SD)	–	1.46 (0–3, SD = 1.29)
DMDD diagnosis, % (*n*)	–	22.6% (26)
MDD diagnosis, % (*n*)	–	4.9% (6)
Anxiety disorder, % (*n*)	–	22.8% (25)

*ADHD* attention-deficit/hyperactivity disorder, *DMDD* disruptive mood dysregulation disorder, *MDD* major depressive disorder, *Anxiety disorder* generalised anxiety disorder or separation anxiety disorder

^a^Number available for each variable ranged from 181 to 249

^b^Number available for each variable ranged from 113 to 124

### Descriptives

Irritability was a common symptom: at baseline (*n *= 696), the mean CAPA irritability symptom score was 2.19 (range 0–3, SD = 1.0), with 91% of the sample reporting at least one irritable symptom. A total of 31% of the sample met diagnostic criteria for DMDD. At follow-up (*n *= 124), the mean DAWBA irritability symptom score was 1.46 (range 0–3, SD = 1.3), with 64% of the sample reporting at least one irritable symptom, and 23% meeting diagnostic criteria for DMDD.

Depression symptoms at follow-up (*n *= 249) were also common: the mean total parent-rated MFQ score was 24.4 (range 0–68, SD = 15.4), with 54.3% of the sample scoring above the clinical cut point of ≥ 21 for parent-rated depression.

### Irritability at baseline and depression symptoms at follow-up (questionnaire sample)

Baseline irritability scores and DMDD diagnosis at baseline were both associated with total parent-rated MFQ score at follow-up, controlling for child age, gender and baseline depression (irritability score: unstandardised *B *= 2.20, 95% CI 0.16, 4.28, standardised beta = 0.14, *p *= 0.035; DMDD: unstandardised *B *= 4.53, 95% CI = 0.53, 8.52, standardised beta = 0.15, *p *= 0.027) (Table 3: model 2, and Table 4: model 2). However, using the MFQ binary measure as an outcome, associations were only present for unadjusted models (see Supplementary Tables S3 and S4).

**Table 3:** Association between irritability score at baseline and parent-rated total MFQ score at follow-up

	Outcome: MFQ total (T2)		
	*B* (95% CI)	Beta (standardised)	*p* value
Model 1: irritable score (T1): unadjusted	3.28 (1.25, 5.32)	0.21	0.002
Model 2: irritable score (T1): controlling for baseline age, gender, depression symptoms	2.20 (0.16, 4.25)	0.14	0.035
Model 3: irritable score (T1): controlling for baseline age, gender, depression symptoms, ADHD medication	2.22 (0.16, 4.28)	0.14	0.035
Model 4: irritable score (T1): controlling for baseline age, gender, depression symptoms and anxiety	1.82 (− 0.24, 3.87)	0.11	0.082
Model 5: irritable score (T1): controlling for baseline age, gender, depression symptoms and ADHD symptoms	1.72 (− 0.39, 3.83)	0.11	0.110
Model 6: irritable score (T1): controlling for baseline age, gender, depression symptoms, ADHD medication, anxiety and ADHD symptoms	1.38 (− 0.76, 3.51)	0.09	0.206

*N* for analysis = 232

*MFQ* Mood and Feelings Questionnaire, *ADHD* attention-deficit/hyperactivity disorder, *T1* at time 1, *T2* at time 2, *B* unstandardised *B* coefficient (*B* is the unit increase in MFQ score for every unit increase in irritable score), *Beta* standardised beta coefficient (Beta is the increase in standard deviations of MFQ score for every standard deviation increase in irritable score)

**Table 4:** Association between DMDD at baseline and parent-rated total MFQ score at follow-up

	Outcome: MFQ total (T2)		
	*B* (95% CI)	Beta (standardised)	*p* value
Model 1: DMDD (T1): unadjusted	6.52 (2.50, 10.53)	0.21	0.002
Model 2: DMDD (T1): controlling for baseline age, gender, depression symptoms	4.53 (0.53, 8.52)	0.15	0.027
Model 3: DMDD (T1): controlling for baseline age, gender, depression symptoms, ADHD medication	4.52 (0.51, 8.53)	0.15	0.027
Model 4: DMDD (T1): controlling for baseline age, gender, depression symptoms and anxiety	3.97 (− 0.019, 7.96)	0.13	0.051
Model 5: DMDD (T1): controlling for baseline age, gender, depression symptoms and ADHD symptoms	3.81 (− 0.25, 7.87)	0.12	0.066
Model 6: DMDD (T1): controlling for baseline age, gender, depression symptoms, ADHD medication, anxiety and ADHD symptoms	3.32 (− 0.74, 7.38)	0.11	0.108

*N* for analysis = 224

*MFQ* Mood and Feelings Questionnaire, *DMDD* disruptive mood dysregulation disorder, *ADHD* attention-deficit/hyperactivity disorder, *T1* at time 1, *T2* at time 2, *B* unstandardised *B* coefficient (*B* is the difference in MFQ score at follow-up in those with DMDD compared to those without DMDD), *Beta* standardised beta coefficient (Beta is the standard deviation unit difference in MFQ score between those with DMDD and those without DMDD)

### Persistent irritability and depression symptoms at follow-up (interview sample)

A total of 63% (71/112) of those with an irritability score of ≥ 1 at baseline continued to have an irritability score of ≥ 1 at follow-up. 37% (19/51) of those who had DMDD at baseline continued to have DMDD at follow-up. Those with persistent irritability (score of ≥ 1 at baseline and follow-up) had higher mean parent-rated MFQ total scores at follow-up compared to those with remitted irritability (27.8 vs 17.1, *t *= − 3.8, *p *< 0.001). Persistent irritability (vs remitted irritability) was associated with total parent-rated MFQ score at follow-up, controlling for age, gender and baseline depression symptoms (unstandardised *B *= 11.79, 95% CI = 6.28, 17.30, standardised beta = 0.38, *p *< 0.001) (Table 5: model 2). Using the MFQ binary measure as an outcome, associations were consistent (OR 6.35, 95% CI 2.41, 16.73, *p *< 0.001) (see Supplementary Table S5).

**Table 5:** Association between persistent irritability and parent-rated total MFQ score at follow-up

	Outcome: MFQ total (T2)		
	*B* (95% CI)	Beta (standardised)	*p* value
Model 1: persistent irritability: unadjusted	10.49 (4.86, 16.12)	0.34	<0.001
Model 2: persistent irritability: controlling for baseline age, gender, depression symptoms	11.79 (6.28, 17.30)	0.38	<0.001
Model 3: persistent irritability: controlling for baseline age, gender, depression symptoms, ADHD medication	12.06 (6.54, 17.59)	0.39	<0.001
Model 4: persistent irritability: controlling for baseline age, gender, depression symptoms and anxiety	11.23 (5.78, 16.70)	0.36	<0.001
Model 5: persistent irritability: controlling for baseline age, gender, depression symptoms and ADHD symptoms	11.24 (5.68, 16.80)	0.36	<0.001
Model 6: persistent irritability: controlling for baseline age, gender, depression symptoms, ADHD medication, anxiety and ADHD symptoms	10.81 (5.30, 16.33)	0.35	<0.001

*N* for analysis = 107

*MFQ* Mood and Feelings Questionnaire, *ADHD* attention-deficit/hyperactivity disorder, *T1* at time 1, *T2* at time 2, *B* unstandardised B coefficient (*B* is the difference in MFQ score at follow-up in those with persistent irritability compared to those with remitted irritability), *Beta* standardised beta coefficient (Beta is the standard deviation unit difference in MFQ score between those with persistent irritability and those without persistent irritability)

### Sensitivity analyses

#### Controlling for additional covariates

Sensitivity analyses found that associations for baseline irritability symptoms and depression, as well as baseline DMDD and depression attenuated when including additional covariates (no longer reaching statistical significance when including anxiety disorder and ADHD symptoms at baseline) (Table 3: models 4–6 and Table 4: models 4–6). However, the association was robust for persistent irritability after all covariates were added (Table 5: model 6).

#### Removing overlapping item

After removing the irritability item from the MFQ at follow-up, the association between irritable score at baseline and parent-rated MFQ total, controlling for age, gender and baseline depression symptoms was slightly weaker (unstandardised *B *= 2.0, 95% CI − 0.02, 4.01, standardised beta = 0.13, *p *= 0.052). The association between DMDD at baseline and parent-rated MFQ total at follow-up remained similar (unstandardised *B *= 4.28, 95% CI 0.35, 8.21, standardised beta = 0.14, *p *= 0.033), as did the association between persistent irritability and depression symptoms (unstandardised *B *= 11.44, 95% CI = 6.06, 16.81, standardised beta = 0.38, *p *< 0.001).

#### Persistent ADHD

Irritability was found to persist alongside ADHD. Of those who had persistent irritability (*n *= 71), 75% also had persistent ADHD (*n *= 53) (OR 2.3, 95% CI 1.02, 5.21, *p *= 0.045). However, persistent irritability continued to be associated with depression symptoms at follow-up after controlling for persistent ADHD (in the sample that completed follow-up interviews) (unstandardised *B *= 10.85, 95% CI 5.41, 16.29, standardised beta = 0.35, *p *< 0.001).

#### Child self-rated MFQ as an outcome

The associations between irritability score and child self-rated depression symptoms and between persistent irritability and child self-rated depression symptoms were similar to the associations seen using parent-rated depression symptoms as an outcome (Tables S6 and S8, Supplementary Material). However, baseline DMDD diagnosis was not associated with child self-reported MFQ score at follow-up (Table S7).

## Discussion

The main aims of this study were to utilise a longitudinal, clinical ADHD sample to examine whether childhood irritability is associated with later depression symptoms and establish whether persistent irritability accounts for this association.

Our results suggest that childhood irritability at baseline (whether defined as a continuous measure or categorical diagnosis of DMDD) is associated with adolescent depression symptoms at follow-up. These results support findings from the general population which have consistently found a longitudinal association between irritability and depression [10]. They also support cross-sectional findings suggesting that irritability is associated with depression symptoms in those with ADHD [14, 15] and findings from a longitudinal population-based sample suggesting that emotion regulation mediates the association between ADHD symptoms and depression symptoms [16]. However, in the current study, we no longer found strong evidence of an association when either baseline anxiety or baseline ADHD symptoms were included as covariates.

With regard to anxiety as a covariate, one possible explanation is that the current study used the MFQ as a measure of depression symptoms at follow-up, rather than depression diagnosis. Although the MFQ is a widely used depression screening instrument [26], items do overlap with symptoms of anxiety, which may explain why anxiety is an important predictor of MFQ total score. It is also possible that the association between baseline irritability and later depression symptoms is explained by co-occurring anxiety disorder. Irritability is associated with anxiety [10], and anxiety (particularly, generalised anxiety disorder) is closely linked to depression [30]. Therefore, anxiety would be a feasible explanation for any association between irritability and depression. However, both irritability and anxiety have been observed to be important antecedents for adolescent depression in other populations, including those at high familial risk for depression [31].

Importantly, for those with persistent irritability, significantly higher depression symptoms were observed in adolescence, even after including childhood anxiety as a covariate. Similarly, whilst baseline ADHD symptoms impacted on the association between baseline irritability and later depression symptoms, the association between persistent irritability and depression symptoms at follow-up remained when including baseline or persistent ADHD symptoms as a covariate. This suggests that it is the persistence of irritability rather than ADHD symptom severity or ADHD persistence that might be important in contributing risk for depression here.

The association between persistent irritability and depression symptoms also remained when the irritability item was removed from the MFQ at follow-up, suggesting that the high parent-rated MFQ score in those with persistent irritability was not as a result of the irritable item on the MFQ. The finding was also consistent when total parent-rated MFQ score or an MFQ clinical cutoff of ≥ 21 was used as an outcome measure, and also when child self-rated MFQ score was used as the outcome measure. The association between persistent irritability and depression is supported by the evidence from studies examining irritability trajectories. These also show that those with persistently high irritability seem to have more depression and internalising symptoms than those who have high initial irritability that decreases over time [17, 18]. However, what was not clear from the present study is whether it is the persistence of irritability or the presence of irritability in adolescence (i.e. present at follow-up) that is particularly important for conferring risk for depression. Due to the majority of this sample having irritability at baseline, it was not possible to test this here.

Overall, these findings suggest irritability is important in the link between ADHD and depression symptoms, although it may be specifically persistent irritability that is important.

These findings are relevant for clinicians. They suggest that, in those with a diagnosis of ADHD, irritability is important and should be identified and monitored. Those who continue to experience irritability into adolescence may be at particular risk for depression and may be the ones who should be considered as a target for intervention/prevention of depression.

### Limitations

It is important to consider a number of limitations in this study. Firstly, the size of the follow- up sample was relatively small, with only a subset of parents completing follow-up interviews. As a result of this, too few met criteria for a diagnosis of major depressive disorder at follow-up to use this as an outcome measure (although all 6 with MDD at follow-up met criteria for DMDD at baseline). The young age of the follow-up sample (mean= 14.4 years) may have contributed to this, with many not yet reaching the peak age of risk for onset of depression. Follow-up into early adulthood would be helpful in future studies.

Another limitation was that the interview measure used to assess irritability and ADHD at follow-up (DAWBA) differed from that used at baseline (CAPA). The DAWBA was used at follow-up as it is a briefer measure than the CAPA, and was more feasible to complete with the families involved. However, this change in measure across the two time points meant that it was not possible to directly compare prevalence of disorders across time in this sample. Despite this, it is worth noting that when comparisons of DAWBA and CAPA were made [32], no significant differences in the rates of ADHD were found and the majority of those with a DAWBA diagnosis also received a CAPA diagnosis.

There are also limitations with regard to the generalisability of the results. Firstly, it could be argued that this ADHD sample may not be representative due to the study exclusion criteria (any major comorbid neurological disorder, psychosis, Tourette’s syndrome, autism or genetic syndrome). However, despite this, many of the disorders that often co-occur with ADHD were not exclusion criteria for this study, e.g. oppositional defiant disorder, conduct disorder, intellectual disability and specific learning disorders, and despite the exclusion of those with a clinical diagnosis of autism, the sample had high levels of autistic traits [33]. Therefore, we consider this sample to be fairly representative of children from South Wales, UK, who have a diagnosis of ADHD. It is also important to note that these results are relevant only to those with clinically ascertained ADHD. Even so, it could be argued that early intervention and prevention of depression may be most feasible for those who are already known to clinical services.

### Conclusions

This study found that persistent irritability in those with ADHD is associated with depression symptoms in adolescence. This suggests that chronically irritable children with ADHD may be a target group for early intervention and prevention of depression, and that those who remain irritable over time should be monitored most carefully.

## Electronic supplementary material

Below is the link to the electronic supplementary material.

Supplementary material 1 (DOCX 29 kb)
