# Supplementary material for: Irritability in ADHD: association with later depression symptoms
Source: Eur Child Adolesc Psychiatry. 2019 Mar 5;28(10):1375–84. doi: 10.1007/s00787-019-01303-x (PMC6785584; doi:10.1007/s00787-019-01303-x)
Supplement: Supplementary file 1 — Supplementary material 1 (DOCX 29 kb) [file 787_2019_1303_MOESM1_ESM.docx]

**Supplementary material:**

**Tables:**

**Table S1: Defining DMDD using Child and Adolescent Psychiatric Assessment (CAPA) at baseline and the Development and Well Being Assessment (DAWBA) at follow-up**

| **DMDD diagnostic criteria** | **CAPA items used**  **(ODD and Depression sections)** | **DAWBA items used**  **(DMDD section)** |
| --- | --- | --- |
| **Severe temper outbursts** | Fulfilled if “losing temper” or “temper tantrum” items present in the ODD section. | Fulfilled if “temper outburst” triggered very easily compared to others (“a little” or “a lot”). |
| **Temper outbursts inconsistent with development** | Fulfilled if either “losing temper” or “temper tantrum” items present in the ODD section. | Fulfilled if “temper outburst” triggered very easily compared to others (“a little” or “a lot”). |
| **Frequency of temper outbursts ≥ 3 x/week** | Fulfilled if “losing temper” frequency total ≥36, or “temper tantrum” frequency total ≥ 36 (equivalent to the symptom being present on average at least 3 x per week over the 3 month period that the CAPA asks about). | Fulfilled if “temper outburst” occurs ≥ 3 times per week over last 12 months. |
| **Irritable or angry mood**  **(mood between outbursts is persistently irritable or angry most of the day, nearly every day and is observable by others)** | Fulfilled if any of the following items from the depression section of the CAPA have a frequency >45:   - “touchy or easily annoyed”, - “angry or resentful”, - “depressed mood” - “irritable”   (equivalent to the symptom being present on more days than not over the 3 month period the CAPA asks about). | Fulfilled if   - “easily get annoyed, or become irritable or angry”, AND - “get into seriously irritable or angry moods that are stronger and more intense than is usual for others of their age”, AND - when irritable or angry they stay that way “most or all of the day” OR they experience “angry” weeks where they are “irritable or angry for most of the day, nearly every day” AND - “irritable or angry mood” is obvious to most other people. |
| **Temper outbursts and irritable mood present for > 12 months** | Fulfilled if “losing temper” OR “temper tantrum” present ≥ 3 x/ week for >12 months, AND “touchy or easily annoyed” OR “angry or resentful” OR “depressed mood” OR “irritable” symptom present ≥ 3 x/ week for >12 months. | Fulfilled if the longest period without a “temper outburst” in last 12 months is < 3 months, AND the longest period without an “angry week” in the last 12 months is < 3 months. |
| **Symptoms present in at least 2 settings** | Fulfilled if “losing temper” or “temper tantrums” were present in at least 2 of the 3 settings asked about in the CAPA i.e. school, home or elsewhere. | Fulfilled if “temper outburst” AND “irritable or angry mood” present in at least 2 of the 3 settings asked about in the DAWBA i.e. home, classroom, with friends. |
| **Diagnosis not to be made before age 6 or after age 18** | No children in this sample were < 6years or >18 years. | No children with available T2 DAWBA data in this sample were < 6years or >18 years. |
| **Temper outbursts and irritable mood onset <10 years** | Fulfilled if date of onset of required symptoms was before the child aged 10 yrs. | Fulfilled if irritability or temper outbursts began before age 10 years. |

DMDD= Disruptive Mood Dysregulation Disorder. ODD= Oppositional Defiant Disorder.

**Table S2: Comparing baseline characteristics of eligible participants who did not take part in follow-up compared to those who did**

|  | **Did not take part at follow up n=185^a^** | **Did take part at follow up**  **n=249^b^** | **Test statistic,**  **p value** |
| --- | --- | --- | --- |
| **Gender, % male (n)** | 83% (154) | 82% (204) | Chi2=0.13, p=0.721 |
| **Age, in years (n)** | 9.5 (185) | 9.0 (249) | t(432)=2.45, p=0.015 |
| **IQ (n)** | 83 (168) | 85 (228) | t(394)=-1.57, p=0.12 |
| **Income, % < £20,000/yr (n)** | 71% (106) | 62% (133) | Chi2=2.84, p=0.092 |
| **ADHD medication, % (n)** | 76.4% (139) | 77.9% (194) | Chi2=0.14, p=0.707 |
| **ADHD symptom score, mean (n)** | 15.1 (180) | 15.5 (246) | t(424)=-1.47, p=0.143 |
| **Irritability score, mean (n)** | 2.26 (178) | 2.22 (247) | t(423)=0.43, p=0.670 |
| **MDD symptom score, mean (n)** | 1.24 (171) | 1.24 (246) | t(415)=0.03, p=0.98 |
| **DMDD diagnosis, % (n)** | 34.3% (59) | 39% (93) | Chi2=1.04, p=0.308 |
| **Anxiety disorder, % (n)** | 6.5% (11) | 7.9% (19) | Chi2=0.31, p=0.580 |
| **MDD diagnosis, % (n)** | 1.2% (2) | 1.6 % (4) | - |

^a^Number for analysis ranged from 150-185; ^b^Number for analysis ranged from 214-249.

ADHD=Attention-Deficit/Hyperactivity Disorder; DMDD=Disruptive Mood Dysregulation Disorder. MDD=Major Depressive Disorder. Anxiety Disorder includes generalised anxiety disorder or separation anxiety disorder. ADHD symptom score, irritability score and MDD symptom score made using the CAPA. DMDD, anxiety disorder and MDD diagnoses made using the CAPA, based on DSM-5 diagnostic criteria.

**Table S3: Examining the association between baseline irritability score and depression symptoms at follow up, using parent-rated MFQ score of ≥ 21 as a binary outcome**

|  | **OR (95% CI)** | **P value** |
| --- | --- | --- |
| **Model 1: Irritable score (T1):** unadjusted | 1.40 (1.10, 1.86) | 0.018 |
| **Model 2: Irritable score (T1):** controlling for baseline age, gender, depression symptoms | 1.24 (0.92, 1.67) | 0.154 |
| **Model 3: Irritable score (T1):** controlling for baseline age, gender, depression symptoms, ADHD medication | 1.25 (0.93, 1.69) | 0.142 |
| **Model 4: Irritable score (T1):** controlling for baseline age, gender, depression symptoms and anxiety | 1.18 (0.88, 1.60) | 0.272 |
| **Model 5: Irritable score (T1):** controlling for baseline age, gender, depression symptoms and ADHD symptoms | 1.13 (0.83, 1.55) | 0.433 |
| **Model 6: Irritable score (T1):** controlling for baseline age, gender, depression symptoms, ADHD medication, anxiety and ADHD symptoms | 1.09 (0.79, 1.50) | 0.594 |

N for analysis= 232. MFQ=Mood and Feelings Questionnaire. ADHD=Attention/Deficit-Hyperactivity Disorder. OR=Odds Ratio.

**Table S4: Examining the association between baseline DMDD and depression symptoms at follow up, using parent-rated MFQ score of ≥ 21 as a binary outcome**

|  | **OR (95% CI)** | **P value** |
| --- | --- | --- |
| **Model 1: DMDD (T1):** unadjusted | 2.23 (1.28, 3.89) | 0.005 |
| **Model 2: DMDD (T1):** controlling for baseline age, gender, depression symptoms | 1.75 (0.97, 3.17) | 0.065 |
| **Model 3: DMDD (T1):** controlling for baseline age, gender, depression symptoms, ADHD medication | 1.75 (0.97, 3.17) | 0.065 |
| **Model 4: DMDD (T1):** controlling for baseline age, gender, depression symptoms and anxiety | 1.68 (0.92, 3.05) | 0.090 |
| **Model 5: DMDD (T1):** controlling for baseline age, gender, depression symptoms and ADHD symptoms | 1.53 (0.83, 2.81) | 0.170 |
| **Model 6: DMDD (T1):** controlling for baseline age, gender, depression symptoms, ADHD medication, anxiety and ADHD symptoms | 1.48 (0.80, 2.74) | 0.207 |

N for analysis=224. DMDD=Disruptive Mood Dysregulation Disorder. MFQ=Mood and Feelings Questionnaire. ADHD=Attention/Deficit-Hyperactivity Disorder. OR=Odds Ratio.

**Table S5: Examining the association between persistent irritability and depression symptoms at follow up, using parent-rated MFQ score of ≥ 21 as a binary outcome**

|  | **OR (95% CI)** | **P value** |
| --- | --- | --- |
| **Model 1:persistent irritability:** unadjusted | 4.70 (2.01, 10.99) | <0.001 |
| **Model 2: persistent irritability:** controlling for baseline age, gender, depression symptoms | 6.35 (2.41, 16.73) | <0.001 |
| **Model 3: persistent irritability:** controlling for baseline age, gender, depression symptoms, ADHD medication | 6.62 (2.49, 17.58) | <0.001 |
| **Model 4: persistent irritability:** controlling for baseline age, gender, depression symptoms and anxiety | 6.03 (2.27, 16.02) | <0.001 |
| **Model 5: persistent irritability:** controlling for baseline age, gender, depression symptoms and ADHD symptoms | 6.01 (2.26, 15.95) | <0.001 |
| **Model 6: persistent irritability:** controlling for baseline age, gender, depression symptoms, ADHD medication, anxiety and ADHD symptoms | 5.78 (2.13, 15.69) | 0.001 |

N for analysis=107. MFQ=Mood and Feelings Questionnaire. ADHD=Attention/Deficit-Hyperactivity Disorder. OR=Odds Ratio.

**Child reported MFQ as an outcome measure**

**Table S6: Association between irritability score at baseline and child-rated total MFQ score at follow up**

|  | **Outcome: MFQ total (T2)** | | |
| --- | --- | --- | --- |
|  | **B**  **(95% CI)** | **Beta (standardized)** | **P value** |
| **Model 1: Irritable score (T1):** unadjusted | 3.39  (0.80, 5.97) | 0.20 | 0.011 |
| **Model 2: Irritable score (T1):** controlling baseline age, gender, depression symptoms | 3.60  (0.93, 6.27) | 0.21 | 0.008 |
| **Model 3: Irritable score (T1):** controlling for baseline age, gender, depression symptoms, ADHD medication | 3.60  (0.92, 6.28) | 0.21 | 0.009 |
| **Model 4: Irritable score (T1):** controlling for baseline age, gender, depression symptoms and anxiety | 3.43  (0.73, 6.13) | 0.20 | 0.013 |
| **Model 5: Irritable score (T1):** controlling for baseline age, gender, depression symptoms and ADHD symptoms | 2.76  (-0.001, 5.51) | 0.16 | 0.050 |
| **Model 6: Irritable score (T1):** controlling for baseline age, gender, depression symptoms, ADHD medication and anxiety and ADHD symptoms | 2.61  (-0.18, 5.40) | 0.15 | 0.067 |

N for analysis=163. MFQ=Mood and Feelings Questionnaire. ADHD=Attention/Deficit-Hyperactivity Disorder. T1=at time 1. T2=at time 2. B=unstandardised B coefficient (B is the unit increase in MFQ score for every unit increase in irritable score). Beta=standardized Beta coefficient (Beta is the increase in standard deviations of MFQ score for every standard deviation increase in irritable score).

**Table S7: Association between DMDD at baseline and child-rated total MFQ score at follow up**

|  | **Outcome: MFQ total (T2)** | | |
| --- | --- | --- | --- |
|  | **B**  **(95% CI)** | **Beta (standardized)** | **P value** |
| **Model 1: DMDD (T1):** unadjusted | 3.50  (-1.43, 8.42) | 0.11 | 0.163 |
| **Model 2: DMDD (T1):** controlling for baseline age, gender, depression symptoms | 3.92  (-1.11, 8.95) | 0.13 | 0.126 |
| **Model 3: DMDD (T1):** controlling for baseline age, gender, depression symptoms, ADHD medication | 3.93  (-1.11, 8.98) | 0.13 | 0.126 |
| **Model 4: DMDD (T1):** controlling for baseline age, gender, depression symptoms and anxiety | 3.51  (-1.59, 8.61) | 0.11 | 0.176 |
| **Model 5: DMDD (T1):** controlling for baseline age, gender, depression symptoms and ADHD symptoms | 2.33  (-2.79, 7.45) | 0.07 | 0.370 |
| **Model 6: DMDD (T1):** controlling for baseline age, gender, depression symptoms, ADHD medication, anxiety and ADHD symptoms | 1.98  (-3.22, 7.18) | 0.06 | 0.453 |

N for analysis=158. MFQ=Mood and Feelings Questionnaire. DMDD=Disruptive Mood Dysregulation Disorder. ADHD=Attention/Deficit-Hyperactivity Disorder. T1=at time 1. T2=at time 2. B=unstandardized B coefficient (B is the difference in MFQ score at follow up in those with DMDD compared to those without DMDD). Beta=standardized Beta coefficient (Beta is the standard deviation unit difference in MFQ score between those with DMDD and those without DMDD).

**Table S8: Association between persistent irritability and child-rated total MFQ score at follow up**

|  | **Outcome: MFQ total (T2)** | | |
| --- | --- | --- | --- |
|  | **B**  **(95% CI)** | **Beta (standardized)** | **P value** |
| **Model 1: persistent irritability:** unadjusted | 9.55  (3.58, 15.5) | 0.32 | 0.002 |
| **Model 2: persistent irritability:** controlling baseline age, gender, depression symptoms | 10.46  (4.14, 16.79) | 0.35 | 0.001 |
| **Model 3: persistent irritability:** controlling for baseline age, gender, depression symptoms, ADHD medication | 10.74  (4.34, 17.13) | 0.36 | 0.001 |
| **Model 4: persistent irritability:** controlling for baseline age, gender, depression symptoms and anxiety | 10.40  (4.01, 16.78) | 0.35 | 0.002 |
| **Model 5: persistent irritability:** controlling for baseline age, gender, depression symptoms and ADHD symptoms | 10.04  (3.63, 16.45) | 0.34 | 0.003 |
| **Model 6: persistent irritability:** controlling for baseline age, gender, depression symptoms, ADHD medication, anxiety and ADHD symptoms | 10.21  (3.65, 16.77) | 0.34 | 0.003 |

N for analysis=91. MFQ=Mood and Feelings Questionnaire. ADHD=Attention/Deficit-Hyperactivity Disorder. T1=at time 1. T2=at time 2. B=unstandardized B coefficient (B is the difference in MFQ score at follow up in those with persistent irritability compared to those with remitted irritability). Beta=standardized Beta coefficient (Beta is the standard deviation unit difference in MFQ score between those with persistent irritability and those without persistent irritability).
